# Effect of Exchangeable Ions in Natural and Modified Zeolites on Ag Content, Ag Nanoparticle Formation and Their Antibacterial Activity

**DOI:** 10.3390/ma14154153

**Published:** 2021-07-26

**Authors:** Neli Mintcheva, Marinela Panayotova, Gospodinka Gicheva, Orlin Gemishev, Georgy Tyuliev

**Affiliations:** 1 Department of Chemistry, University of Mining and Geology, 1700 Sofia, Bulgaria; marichim@mgu.bg (M.P.); g.gicheva@mgu.bg (G.G.); 2 Faculty of Biology, Sofia University “St. Kliment Ohridski”, 1000 Sofia, Bulgaria; o.gemishev@uni-sofia.bg; 3 Institute of Catalysis, Bulgarian Academy of Sciences, 1113 Sofia, Bulgaria; tyuliev@ic.bas.bg

**Keywords:** silver nanoparticles, natural zeolite, modified zeolites, clinoptilolite, nanocomposites, AgNPs-zeolite, antibacterial properties, TEM, XPS

## Abstract

To broaden the application of silver nanoparticles (AgNPs), which are well-known antibacterial agents, they are supported on different substrates to prevent aggregation, increase their surface area and antibacterial efficiency, and to be separated from the system more effectively at the end of treatment. To produce nanocomposites that consist of silver nanoparticles on natural and modified zeolites, silver ions (Ag^+^) were loaded onto zeolite (natural, Na-modified, H-modified) and then thermally reduced to AgNPs. The effect of the exchangeable cations in zeolite on Ag^+^ uptake, AgNPs formation, size and morphology was investigated by the TEM, SEM, EDX, XPS, UV-vis, XRD and BET methods. The silver amount in the nanocomposites decreased in the following order Na-modified zeolite > natural zeolite > H-modified zeolite. Microscopic techniques showed formation of AgNPs of 1–14 nm on natural and Na-modified zeolite, while the diameter of metal particles on H-modified zeolite was 12–42 nm. Diffuse reflectance UV-vis and XPS methods revealed the presence of both silver ions and AgNPs in the materials indicating that partial reduction of Ag^+^ ions took place upon heating at 400 °C in air. Additionally, antibacterial properties of the nanocomposites were tested against *Escherichia coli*, and it was found that Ag–containing composites originating from the Na-modified zeolite demonstrated the highest activity.

## 1. Introduction

Zeolites are hydrated crystalline aluminosilicates, containing exchangeable cations (Na^+^, K^+^, Ca^2+^, Mg^2+^) which compensate for the negative charge in the Al–O–Si network. Clinoptilolite is highly abundant mineral in many geographic regions. It is isostructural to heulandite, and both belong to the framework type HEU [1,2]. The SiO_4_ and AlO_4_ tetrahedral units, connected via shared oxygen atoms, form the three-dimensional structure of clinoptilolite, which is characterized by three types of mutually crossing channels. Two of them, channel A and channel B, corresponding to the 10-membered rings with the dimension 3.1 × 7.5 Å and the 8-membered rings having dimension 3.6 × 4.6 Å, are along the *c* axis (001). Channel C is along the *a* axis (100) and formed by 8-member rings with size 2.8 × 4.7 Å [1]. Thus, two types of cages are formed by the intersection of channels A and C, and the intersection of channels C and B. It was determined crystallographically that in the framework there were four main cation positions, denoted as M1, M2, M3 and M4 [3]. The M1 and M2 were assigned to be positions for Na^+^ and Ca^2+^ ions in channel A and channel B, respectively. The M3 place was for K^+^ ion in channel C, while M4 was for Mg^2+^ in channel A. Each cation was surrounded by a certain number of O atoms from the framework and H_2_O molecules, and its coordination mode and location influenced the exchange capabilities of the metal ion [3]. The ion exchange was also affected by cation charge, size and ionization potential [4].

The typical ion-exchange properties of natural zeolites derive from a facile exchange of the aforementioned cations with many other metal cations. Natural zeolites are well-known and low-cost exchangers for heavy metal removal from waste waters [5]. Furthermore, based on the selective adsorption and natural availability of zeolites, investigations have been carried out for many years to explore their application in water treatment [6], agriculture [7], pharmaceuticals [8], and drug delivery [9] and to enhance their properties, such as adsorbing cations [10,11], anions [12], and gaseous molecules [13] as well as catalytic activity [14]. The sorption properties of natural zeolites can be tailored by modification using chemical reagents (acids, bases, salts) that change the cation transfer ability, porosity and even Si/Al ratio in the zeolite framework that alters the ion-exchange capacity [15,16]. In this investigation we explore the consequence of pretreating natural zeolite with HCl and NaCl on the silver uptake and the following reduction to metallic Ag.

Silver ions are among the cations that can be easily exchanged to govern the antibacterial properties of modified zeolites and to widen the application of composites. The Ag–modified zeolites could be desired for pharmaceutical use [17], water purification [18], food packaging and preservation [19] because of their proven bactericidal efficiency, safety and thermal stability mainly due to the antimicrobial action of silver and the stable and inert alumosilicate support that allows the gradual and continuous release of Ag^+^ into the medium [20]. Many researchers have shown the antibacterial properties of Ag–zeolite composites by using both natural and synthetic zeolites [21,22,23]. Not only Ag^+^-exchanged zeolites were applied but also those having Ag_2_O or AgNPs on the zeolite surface were investigated for the effectiveness and mechanism of action of Ag-loaded composites [21,23,24,25,26].

The methods, which have been developed to stabilize Ag on the nanoscale, comprise a silver ion-exchange on zeolite followed by thermal or chemical reduction to clusters and nanoparticles. Concepción-Rosabal et al. heated silver-modified natural and synthetic clinoptilolite in a hydrogen atmosphere at different temperatures to produce AgNPs of various dimensions [27]. Flores-López et al. achieved AgNPs by heating silver-exchanged natural chabazite in air at 400 °C for 1 h [28]. Other authors used chemical reducing agents such as sodium borohydride to reduce Ag^+^ supported on zeolite to AgNPs-containing nanocomposites [21,26] or trisodium citrate solution to treat silver-ion-immobilized ZSM-5 zeolite [29].

However, to the best of our knowledge little research has been done on the silver content and silver oxidation state stabilized in natural zeolite based on the exchangeable ions in the precursors and their relationship with the antibacterial properties of nanocomposites. The thermal stability of silver forms in silver-modified natural and synthetic clinoptilolite were studied by Concepción-Rosabal et al. [27]. The nature of the exchangeable cations (H+, Na^+^, Cu^+^/Cu^2+^, Ag^+^, Pb^2+^) and the effect of the Si/Al ratio in faujasite zeolites on the silver oxidation state and the CH_3_I adsorption were reported very recently [30]. Hence, more investigation and experimental evidence are needed for silver species in silver–zeolite composites to ensuring the directed synthesis of AgNPs-composites with specific functionalities and applications.

In this study, we intended to modify natural zeolite to prepare silver-containing nanocomposites and to test them for antibacterial activity against *Escherichia coli*. The structural and elemental analyses of as-prepared materials were conducted by advanced methods to evaluate the effect of exchangeable cations in natural and modified zeolites on the metal loading and morphology of AgNPs incorporated in the zeolite. Microbiological experiments were run to explore the effectiveness of new nanocomposites as antibacterial agents and to select the most efficient one.

## 2. Materials and Methods

### 2.1. Material Preparation

A natural zeolite from the region of East Rhodopes, Bulgaria, was used for all experiments. The selected fraction of raw material with size 0.09–0.325 mm was rigorously washed, dried and analyzed. The purified material contained 76% clinoptilolite-Ca [Ca_3_(Si_30_Al_6_)O_72_·20H_2_O] and 24% Barrerite [(Na,K,Ca_0.5_)_2_(Al_2_Si_7_O_18_)·7H_2_O] as per XRD analysis. The classical silicate analysis showed the following composition (in wt%): SiO_2_ 69.25, Al_2_O_3_ 12.96, CaO 3.34, MgO 0.70, K_2_O 2.89, Na_2_O 0.46, Fe_2_O_3_ 0.34, MnO 0.03, TiO_2_ 0.08, P_2_O_3_ < 0.05, SO_3_ < 0.05, LOI 9.82. Modification of natural zeolite in Na-form and H-form was performed by using 2 M NaCl and 10^−4^ M HCl solutions (pH 4.6), respectively, at a solid–liquid ratio of 1:10 and constant stirring at room temperature for 7 days. Then, the washed and dried materials were immersed in 0.1 M solution of AgNO_3_ at pH 6 and solid–liquid ratio of 1:20 and stirred for 4 h. After filtration and thorough washing with distilled water till a negative reaction for Ag^+^ in washings, the materials were dried at 50 °C and heated at 400 °C for 2 h in air. The obtained silver nanocomposites with natural zeolite, Na-modified zeolite and H-modified zeolite were denoted as Ag–Zeo, Ag–NaZeo and Ag–HZeo, respectively. More details about the preparation procedure are described in our previous paper [31]. During the preparation and analysis precautions were taken for the light sensitivity of silver-containing samples.

### 2.2. Material Characterization

The morphology and microstructure of nanocomposites were studied by scanning electron microscopy (SEM) and transmission electron microscopy (TEM). The SEM images were obtained on a JEOL 6390 scanning electron microscope (JEOL, Tokyo, Japan) and TEM images were observed on a JEOL JEM-2100 microscope (JEOL, Tokyo, Japan) with an accelerating voltage of 200 kV. The surface chemical composition of materials was determined by energy dispersive X-ray spectroscopy (EDX) performed on INCA Oxford analyzer (Oxford instruments, Concord, MA, USA) combined with SEM.

The X-ray photoelectron spectroscopy (XPS) was used to analyze the elements and their oxidation states on the surface. The measurements were carried out in the chamber of photoelectron spectrometer Escalab-MkII (VG Scientific, East Grinstead, UK) with a base pressure of 1 × 10^−10^ mbar. The electrons were excited with AlKα radiation (hν = 1486.6 eV) at low power (5 mA/6 kV) of the X-ray source to minimize the effect of irradiation on the sample during data acquisition. The binding energy calibration was done by using the strongest O1s line in the spectra centered at 532.7 eV.

The specific surface area of nanocomposites was determined by using the Brunauer–Emmet–Teller (BET) method on the Quantachrome NOVA 1200e Analyzer (Quantachrome Instruments, Boynton Beach, FL, USA) by sorption of N_2_. Before measurement, the samples were outgassed at 200 °C for 16 h under vacuum. The total pore volume and the average pore diameter were obtained at a relative pressure of p/p_0_ ≈ 0.99 according to the Gurvich rule. The surface area and volume of micropores, and pores distribution were calculated by using *t*-method and the Barrett–Joyner–Halenda (BJH) method, respectively.

To evaluate different silver forms in zeolites, diffuse-reflectance UV–vis spectra were taken on an Evolution 300 UV-Vis Spectrophotometer (Thermo Fisher Scientific, Waltham, MA, USA). Spectralon was used for the background measurements.

The XRD patterns were obtained on Bruker D8 Advance powder diffractometer (Bruker, Karlsruhe, Germany) with Cu Kα radiation and a LynxEye detector (Bruker, Karlsruhe, Germany).

### 2.3. Antibacterial Tests

*Escherichia coli* strain 3398, used in the tests, was pre-grown on a Luria agar at 37.0 ± 0.1 °C for 16 h to obtain cultures in a log phase of growth. For preparation of Peptone water (PW) 10.0 g peptone and 0.5 g NaCl were dissolved in 1 L distilled water. Aliquots of 10 mL of PW were inoculated with *E. coli* (10^5^ CFU/mL). To determine the minimum inhibitory concentration (MIC) of the synthesized Ag–zeolite composites (Ag–Zeo, Ag–NaZeo, Ag–HZeo), the material was added to the inoculated PW at concentrations from 0.2 to 5.0 mg/mL. The samples were cultivated for 24 h at 37.0 ± 0.1 °C. Then 1 mL of the suspension was diluted in sterile distilled water (serially in triplicate) and volumes of 0.1 mL were aseptically inoculated onto the Lysogeny agar (LA)–Luria (0.5 g/L NaCl) plates. The plates were incubated at 37.0 ± 0.1 °C for 24 h. After that the bacterial colonies were counted and the number of viable cells was reported as CFU/mL. As controls were used natural zeolite (from 0.2 to 5.0 mg/mL suspensions in PW) and *E. coli* suspension in PW (without zeolite and nanocomposites added). The controls were treated (dilutions and incubation) like the other samples variants. The antibacterial activity was calculated using the formula:

$$Antibacterial\;activity\;\left(\%\right)=\frac{CFU_{control}-CFU_{Ag\;nanocomposite}}{CFU_{control}}\times 100$$

The lowest nanocomposite concentration that prevents visible growth of *E. coli* is defined as the minimum inhibitory concentration (MIC) which corresponds to nearly 100% antibacterial activity.

## 3. Results and Discussion

The nanocomposites Ag–Zeo, Ag–NaZeo and Ag–HZeo were prepared by heating Ag-exchanged natural, Na- and H-modified zeolites, respectively at 400 °C for 2 h in air. As-prepared materials were characterized by SEM, TEM, EDX, XRD, UV-vis, XPS, BET, and their antibacterial activity towards *E. coli* was tested.

### 3.1. Characterization of Nanocomposites

#### 3.1.1. SEM and Morphology

**Figure 1:**
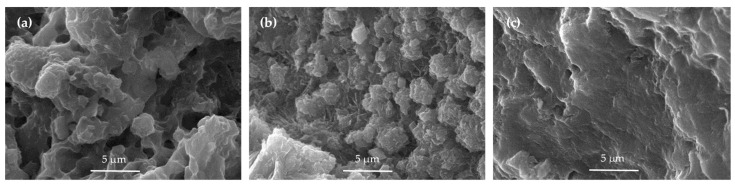
SEM micrographs of samples: (**a**) Ag–Zeo, (**b**) Ag–NaZeo and (**c**) Ag–HZeo.

The morphology of the prepared samples was examined by SEM and the selected micrographs are presented in Figure 1 and Figure S1. Irregular shapes, voids and aggregates of plate-like, layered particles are typical for the silver nanocomposites. The samples Ag–Zeo and Ag–NaZeo show rough surface with many clusters (Figure 1a,b). After modification with HCl and subsequent Ag–loading, the morphology of Ag–HZeo was changed and the surface was observed to be smoother (Figure 1c), most probably because of the partial solubility of the zeolite in an acidic solution, which was confirmed by the chemical composition of H-form (see Section 3.1.2).

#### 3.1.2. EDX and Element Composition

The chemical composition of silver–zeolite materials determined by EDX analysis showed an increase in the Si/Al ratio in Ag–HZeo and a decrease in the amount of exchangeable cations of K, Ca, Mg after treatment of natural zeolite in an acidic solution. The Si/Al ratio was 5.77 and 5.73 for Ag–Zeo and Ag–NaZeo, respectively, while that of Ag–HZeo changed to 15.2 after intense dealumination (Table 1).

The H-zeolite form was found to have the lowest exchange rate of Ag^+^ ions, as would be expected for dealuminated zeolites [30], so that Ag–HZeo possessed the lowest Ag content (2.2%) among the nanocomposites. The Ag uptake on natural zeolite in Ag–Zeo was 10.4 wt.% (Table 1). The pretreatment of natural zeolite with NaCl enriched it with Na ions, thus Na-modified zeolite possessed a higher amount of easy-removable Na^+^ ions resulting in an increased silver exchange and Ag upload [32]. This was the reason for the highest silver load (14.6 wt.%) found in Ag–NaZeo. Na-modified clinoptilolite was reported to be selective for Ag^+^ ions with the standard free energy of exchange—6.0 kJ/equiv [33]. Lihareva et al. also found higher sorption of Ag^+^ ions on Na-exchanged clinoptilolite than on a natural one [34].

In our measurements, the Na^+^ ion was not detected by EDX, implying it was entirely exchanged by Ag^+^ ions. The other exchangeable ions seemed to show different content in the samples resulting from the pre-treatment of natural zeolite. In Ag–NaZeo, the amount of Ca, Mg, Fe ions were lower in comparison with Ag–Zeo, indicating an intensive ion-exchange. In Ag–HZeo, Ca and Fe were very close to Ag–Zeo, while the percent of K and Mg was lower.

#### 3.1.3. BET and Specific Surface Area

The nitrogen adsorption–desorption isotherms for all samples were typical for mesoporous materials and displayed the H3 hysteresis loops, indicating that the materials consisted of agglomerates from flat particles [35,36] (Figure S2), which was in line with the SEM observations, as can be seen in Figure 1a,b and Figure S1. In case of the sample Ag–HZeo, the hysteresis was extended to the lowest attainable pressure, most probably due to irreversible gas adsorption or the swelling of the structure and enlargement of pore volume (Figure S2c). The specific surface area and pore size (Table 2) of natural zeolite were similar to what was previously reported for clinoptilolite from other regions [37,38,39]. The micropore surface area and volume were low (7 m^2^/g and 0.003 cm^3^/g, respectively) which may be assigned to blocked zeolite channels by the exchangeable cations. Similar values (5.6 m^2^/g and 0.003 cm^3^/g, respectively) were found for the clinoptilolite from Slovakia [39]. At the same time, its specific surface area (33.5 m^2^/g) and external surface area (27.9 m^2^/g) were higher than those of the Bulgarian clinoptilolite, which was not surprising bearing in mind different phase composition of natural products [39]. The natural zeolite-specific surface area (S_BET_) and pore parameters were slightly affected by the Na-ion-exchange, in contrast to the changes induced by H-modification. The specific surface area of H-modified zeolite (S_BET_ 184 m^2^/g) was significantly higher than that of the natural and Na-modified zeolite (S_BET_ 19 and 18 m^2^/g, respectively). The pore volume (V_total_ 0.17 cm^3^/g) increased and the average pore diameter (D_av_ 3.7 nm) decreased in H–Zeo compared with that of Zeo (V_total_ 0.08 cm^3^/g and D_av_ 16 nm) and Na–Zeo (V_total_ 0.10 cm^3^/g and D_av_ 21 nm). After acid treatment, the total pore volume doubled, while total micropore volume and micropore surface area increase 20 times due to the exchange of cations in the pores and the dealumination of the zeolite framework (Table 2). A progressive increase in specific surface area and pore volume and a decrease of pore width by increasing of acid concentration in the treatment of natural zeolite were observed by Wang et al. [40]. A similar trend in porosity was observed for silver–zeolite composites, as Ag–HZeo showed higher S_BET_ (90 m^2^/g) than did the Ag–Zeo or Ag–NaZeo samples (both 15 m^2^/g). Similarly, the AgNPs loading on zeolites led to a decrease in S_BET_ and total pore volume, as well as an increase in average pore diameter in comparison with silver-free precursors, which is attributed to the position of AgNPs in the zeolites’ cavities. Such changes in specific surface area and pore dimensions were also observed with the increasing in metallic Ag uptake in Ag–ZSM-5 nanocomposites [29]. Interestingly, the surface area and volume of micropores in the samples Ag–Zeo and Ag–NaZeo were calculated to be zero, indicating that those sites were blocked with tiny silver clusters or nanoparticles. These results are consistent with the TEM images where nanoparticles as small as 1–2 nm were observed.

The average pore diameter in both H-modified zeolite and Ag–HZeo was smaller than for other samples, indicating that significant changes in the zeolite structure resulted from the acidic pre-treatment of natural zeolite.

#### 3.1.4. TEM and Size Distribution

**Figure 2:**
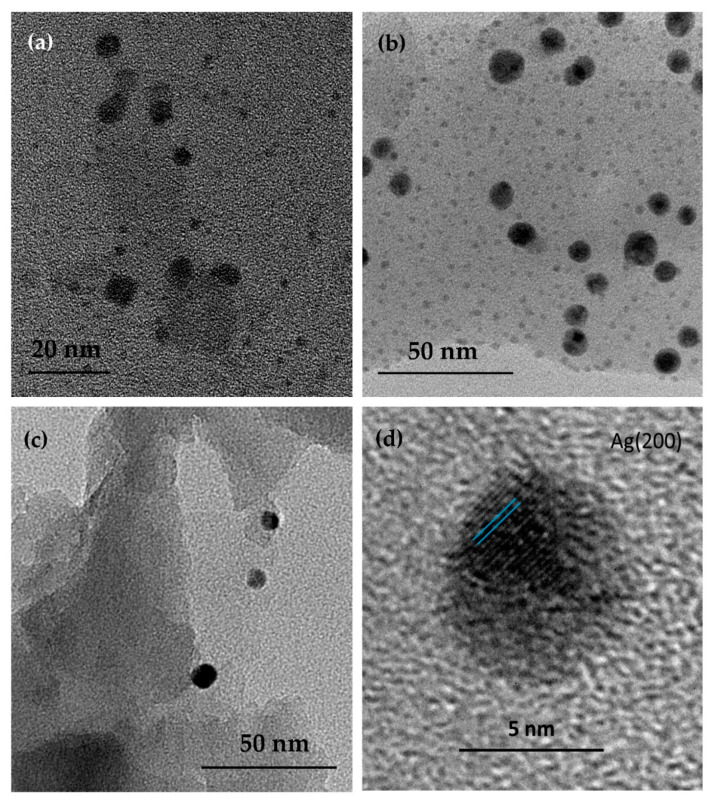
TEM images at high magnification of silver–zeolite composites: (**a**) Ag–Zeo, (**b**) Ag–NaZeo, (**c**) Ag–HZeo, (**d**) HRTEM of AgNP.

**Figure 3:**
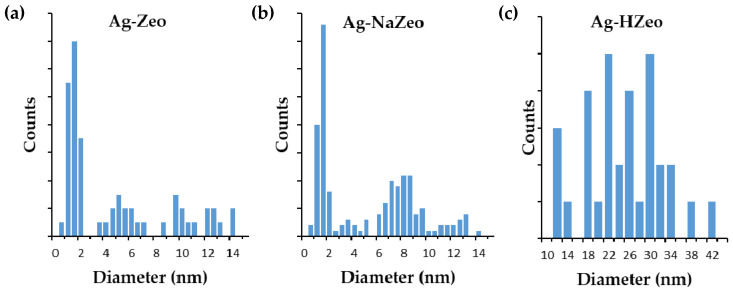
AgNP size distribution in silver–zeolite composites. (**a**) Ag–Zeo (**b**) Ag–NaZeo (**c**) Ag–HZeo.

The TEM images of silver-zeolite composites are shown in Figure 2. In all cases sphere-like AgNPs in a wide range of sizes was observed. To evaluate the effect of exchangeable ions in zeolite precursors on the size and distribution of AgNPs in nanocomposites, TEM images were used to determine the diameter of AgNPs. The particle size distributions, shown in Figure 3a,b, reveal two types sub-size NPs in Ag–Zeo and Ag–NaZeo samples. The small particles with a diameter of 1–2 nm were found in both materials. Such NPs are too large to be located in the zeolite’s cages along the channels and can be attributed to the formation of AgNPs on the outer surface of the zeolite crystals and evenly distributed within the structure.

The second fraction of AgNPs with a mean size of 5 and 8 nm for Ag–Zeo and Ag–NaZeo, respectively, as well as larger AgNPs with diameter in the range of 10–14 nm were also observed in both samples. Most probably these particles were located on the crystals’ surface and in the mesopores of clinoptilolite. The size difference in the samples Ag–Zeo and Ag–NaZeo could be explained by the presence of different amounts and types of exchangeable cations in the zeolite channels of both materials, and this affected the migration of silver ions and the aggregation of Ag–clusters in the process of formatting AgNPs [41].

The determined weight percentage of Ag in Ag–Zeo and Ag–NaZeo is 10.4 and 14.6%, respectively (Table 1), indicating a high degree of Ag^+^ ion-exchange in natural zeolite and Na-modified zeolite, hence we suggest that the silver ions are regularly spread within the zeolite framework of the precursors and are available for further reduction while being immobilized by alumosilicate crystals. Once the Ag^+^ ions are accommodated in the zeolite channels, they undergo partial thermal reduction during heating at 400 °C for 2 h in air.

Bartolomeu et al. also found formations of AgNPs 3–4 nm in size after the calcination of Ag–zeolite L and proposed Ag^+^ reduction under oxidative conditions [42]. Such a process takes place via stepwise growth of Ag–clusters in zeolite structure (Ag→Ag_2_→Ag_3_→…→Ag_n_) and further aggregation to AgNPs, the size of which depends on the zeolite type, type of exchangeable ions and reaction conditions [41]. After heating in air, other authors observed AgNPs with an average diameter of 8 nm on the surface of a chabazite microcrystals [28] or a bimodal size distribution of AgNPs (3–5 and 20–25 nm) in natural clinoptilolite [43]. Azambre et al. found the co-existence of Ag_n_^+^ and Ag_m_^0^ clusters with Ag^+^ cations and suggested an auto-reduction process for the formation of Ag–clusters in silver-exchanged Y zeolites after calcination [30].

Concepcion-Rosabal et al. found that after a reduction at 100 °C, in the sample Ag–synthetic clinoptilolite, Ag_2_^+^ and Ag_4_^δ+^ clusters were formed in the clinoptilolite pores, while in the Ag–natural zeolite (containing clinoptilolite, mordenite and other mineral phases), both small Ag_2_^+^–Ag_4_^+^ and larger Ag_8_^0^–Ag_8_^δ+^ clusters were obtained, as the latter were in the large mordenite channels or in the spaces between the zeolite particles. At 400 °C, those clusters became unstable and aggregated to larger NPs 5–15 nm in size on the surface of zeolite crystals for both samples [27]. Our TEM observations were in good agreement with the results reported in [27] and showed that 1–14 nm AgNPs are formed during heating at 400 °C of Ag–exchanged natural and Na-modified clinoptilolite.

In contrast, AgNPs in the Ag–HZeo sample were estimated to be within a size range 12–42 nm and positioned on the external surface of zeolite particles, as can be seen in Figure 2c. Due to a lower amount of exchangeable cations (Ca^2+^, Mg^2+^, Na^+^, K^+^) in the channels of H-modified zeolite, fewer Ag^+^ ions were immobilized on the zeolite. The lower Ag^+^ uptake on the H-modified zeolite led to the formation of fewer AgNPs, as seen in the TEM images. Moreover, the NP size was larger in comparison with that in the Ag–Zeo and Ag–NaZeo samples, most probably due to a higher Si/Al ratio and lower acidity, which favored the reduction of Ag^+^ ions and growth of Ag clusters. Azambre et al. reported that the Si/Al ratio affected the state and distribution of silver in faujasite zeolites because at a low Si/Al ratio (2.5) the finely dispersed Ag^+^ and Ag clusters within the zeolite pores are obtained, while at high ratio (Si/Al = 40) metallic Ag NPs on the external surface were formed [30].

Crystalline AgNPs formed by heating silver-exchanged zeolites were proven by high-resolution TEM. The lattice fringes observed in the HRTEM image (Figure 2d) showed a distance of 0.20 nm, which corresponds to the (200) planes of cubic Ag structure (PDF 004-0783). In addition to this, evidence for crystallographic planes of Ag_2_O crystals was not found.

In line with the TEM observations, the XRD patterns of all samples (shown in Figure S3) did not show any peaks for the crystal phase of metallic Ag or Ag_2_O, which may due to the small AgNPs evenly distributed within the zeolite framework and/or the low fraction of metallic Ag^0^ in the nanocomposites [43]. Overall, the observed XRD peaks correspond to the XRD patterns of Ag-exchanged clinoptilolite (PDF 01-081-8531). The crystallinity of Ag–HZeo decreased after treatment of natural zeolite with HCl and Ag–ion exchange.

#### 3.1.5. Diffuse Reflectance UV-vis Spectroscopy and Silver Forms in Nanocomposites

**Figure 4:**
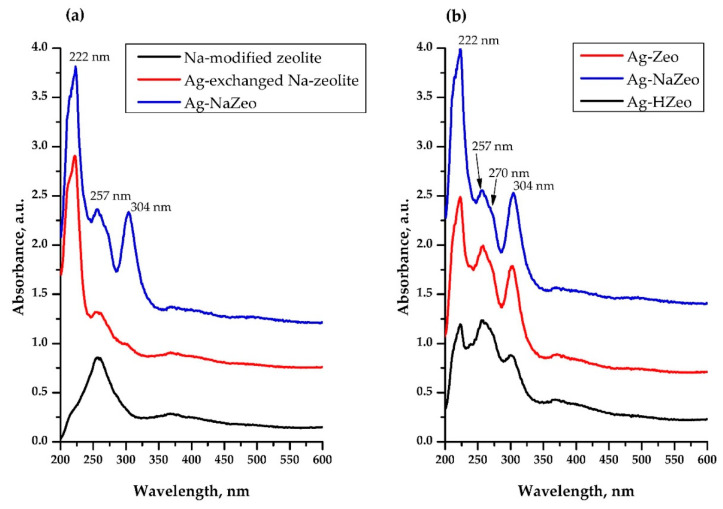
Diffuse Reflectance UV-vis spectra of: (**a**) precursors Na-modified zeolite (black line), Ag–exchanged Na-zeolite (red line), Ag–NaZeo (blue line); (**b**) nanocomposites Ag–HZeo (black line), Ag–Zeo (red line), Ag–NaZeo (blue line).

Diffuse reflectance UV-vis spectra were used to identify Ag species in the nanocomposites. For comparison, in Figure 4a UV-vis spectra of Na-modified zeolite, Ag–exchanged Na-zeolite and Ag–NaZeo composite are shown. Na-modified zeolite exhibited a peak at 257 nm due to the zeolite framework, which was observed in all other samples as well. Such a band was characteristic for zeolites and originated from the charge transfer from oxide ion, O^2−^ to Al^3+^ ion located at specific sites such as defects, corners and surfaces [44,45]. The Ag loading in Na-modified zeolite led to the appearance of the peak at 222 nm, which was assigned to the charge-transfer band of Ag^+^ ions immobilized in the zeolite [28,42,46]. After heating of the latter material, a new peak at 304 nm appeared in the spectrum of the Ag–NaZeo sample and could be assigned to AgNPs. Thus, we can corroborate the TEM finding for the observed metal Ag particles in all samples (Figure 2). Apart from the peak at 304 nm in Figure 4b, a shoulder at 270 nm was also observed in the UV-vis spectra of Ag–Zeo, Ag–NaZeo and Ag–HZeo, which might be assigned to Ag clusters in zeolite channels. Gradually these clusters grew to nanoparticles which appeared at higher wavelength in the UV-vis spectra. Some authors suggested absorption bands for silver clusters (Ag_n_^δ+^ and Ag_m_) in the range of 240–350 nm [30,42,47] and surface plasmon resonance of AgNPs at about 400 nm [21]; for example, the peaks at 300 and 410 nm were assigned to AgNPs in [27]. The same authors reported that the peak observed at 275 nm in the spectrum of Ag–synthetic clinoptilolite was attributed to Ag_4_^δ+^ clusters, while the peaks at 325 and 290 nm were assigned to the Ag_8_^0^ and Ag_8_^δ+^ clusters, respectively [27].

Interestingly, the intensity of the bands at 222 and 304 nm increased in the order Ag–HZeo < Ag–Zeo < Ag–NaZeo (Figure 4b), which accorded with the increase in Ag content found by EDX (Table 1). Thus, the UV-vis spectra of the nanocomposites revealed the presence of different silver species (Ag^+^ ions, Ag clusters and AgNPs), which supported the hypothesis for the thermal reduction of Ag^+^ to AgNPs upon heating in air.

#### 3.1.6. XPS and Surface Chemistry

**Figure 5:**
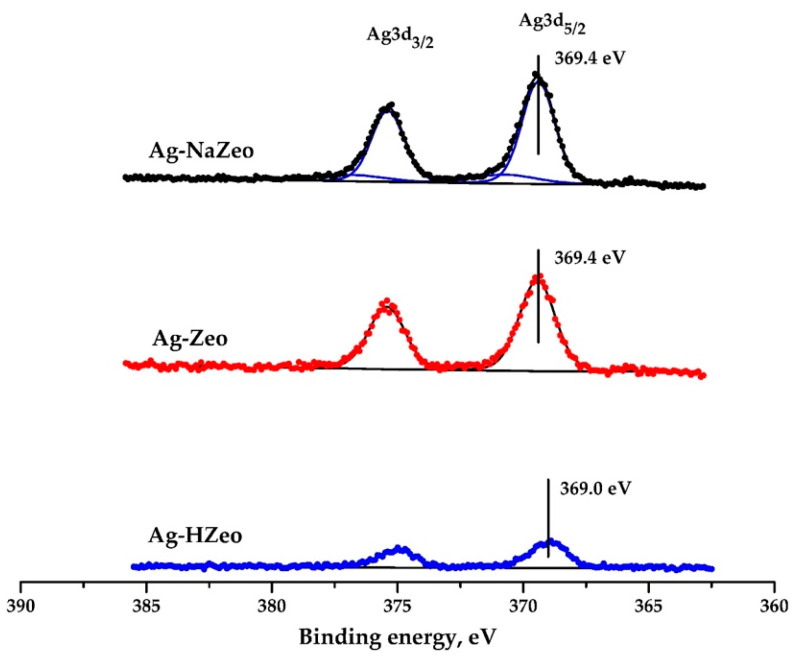
Photoelectron Ag 3d spectra of samples Ag–Zeo, Ag–NaZeo, Ag–HZeo.

The silver oxidation state in the nanocomposites was studied by means of XPS. The doublets Ag3d_5/2_ and Ag3d_3/2_ of the Ag–Zeo, Ag–NaZeo and Ag–HZeo samples are shown in Figure 5 and the values of the XPS parameters are given in Table 3. The binding energy was calibrated with respect to O 1s signal observed as a singlet at 532.7 eV and arose from oxide ions in the zeolite framework. This approach was chosen as an alternative for the reference C 1s peak of adventitious carbon, which was not suitable in the case of poor conductors like zeolites [48]. The Ag 3d peak positions for samples Ag–Zeo and Ag–NaZeo were identical (369.4 and 375.4 eV for Ag3d_5/2_ and Ag3d_3/2_, respectively), but for sample Ag–HZeo the signal was slightly shifted towards lower values (369.0 and 375.0 eV), which might be ascribed to the greater Ag^+^ fraction in the latter nanocomposite. Moreover, the peaks’ intensity increased in the order Ag–HZeo < Ag–Zeo < Ag–NaZeo in accord with the Ag–content in the zeolites (Table 1). Although the observed binding energy for Ag 3d was higher than that reported for metallic Ag (368.3 and 374.3 eV for Ag3d_5/2_ and Ag3d_3/2_) and silver oxides (Ag3d_5/2_ 367.5–368 eV) [49], we assumed the signals of nanocomposites were due to both species Ag(0) and Ag(I) which was supported by the Auger parameter as well. Peaks of Ag3d_5/2_ at 368.5–369.7 eV for Ag–loaded zeolites were also reported in the literature [12,23,50]. The small difference between the binding energies of Ag(0) and Ag(I) and the narrow Ag3d peaks made the assignment difficult and ambiguous. That is why, to confirm the silver species in the nanocomposites, we analyzed Auger spectra (Figure S4) and calculated the Auger parameter (α’), which was the sum of the binding energy of Ag 3d_5/2_ and the kinetic energy of AgM_4_NN [51]. The Auger lines AgM_4_NN and AgM_5_NN were expected at the binding energy of 1129 eV and 1135 eV according to the reference book [49]; however, we observed those transitions at higher energy as wide peaks that were deconvoluted into 4 components originating from two silver species Ag(0) and Ag(I), and the binding energies for AgM_4_NN were estimated to be 1131 eV and 1133 eV for Ag(0) and Ag(I), respectively (Table 3). The Auger parameter for the metallic silver was 726.0 eV, and that of the silver ion, 724.0 eV [51]. The results for the kinetic energy of AgM_4_NN transitions and the corresponding α’ parameters are given in Table 3. The calculated values of the Auger parameter (α’) are 725.0 and 723.0 eV, which were attributed to Ag(0) and Ag(I), respectively [50] showing that silver exists in different oxidation states in the zeolite composites.

### 3.2. Antibacterial Activity of Silver-Zeolite Composites

**Figure 6:**
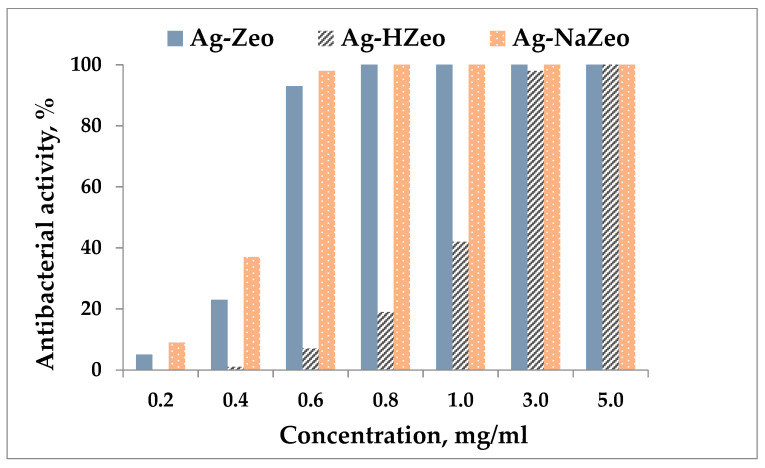
Antibacterial activity against *Escherichia coli* of samples Ag–Zeo, Ag–NaZeo, Ag–HZeo.

The antibacterial activity of nanocomposites was investigated by determining the minimum inhibitory concentration against the Gram-negative bacterium *Escherichia coli* as an indicator strain. The tests were performed with concentrations in the range of 0.2–5.0 mg/mL of Ag–loaded nanocomposites (Ag–Zeo, Ag–NaZeo, Ag–HZeo). The percent antibacterial activity of each nanocomposite was calculated based on a comparison between the bacterial growth in its presence and the control (*E. coli* suspension in PW without any zeolite), which exhibited bacterial growth 10^7^ CFU/mL. The results are presented in Figure 6, where it clearly shows that the antibacterial activity increased along with the concentration of nanocomposite that was proportional to the Ag content in the nanocomposite. The MIC for samples Ag–Zeo and Ag–NaZeo was estimated at 0.8 mg/mL and for Ag–HZeo, 5.0 mg/mL.

The antibacterial effect of the three nanocomposites applied in different concentrations is illustrated in Figures S5–S7. Inoculation of the plates with LA medium was performed with microbial material (*Escherichia coli* 3398) taken from the reaction mixture (without dilution). Figure S5 shows that all nanocomposites at concentration 5.0 mg/mL completely inhibited bacterial growth for 24 h. In Figure S6, one can see that in case of Ag–Zeo and Ag–NaZeo (3.0 mg/mL) no bacterial colonies were observed, while the concentration 3.0 mg/mL of Ag–HZeo was not enough to kill all bacteria, so antibacterial activity of 98% was calculated (Figure 6). Antibacterial effect of both Ag–Zeo and Ag–NaZeo at concentrations of 1.0, 0.8 and 0.6 mg/mL was very similar, as can be seen in Figure S7. However, at lower concentration (<0.6 mg/mL) Ag–Zeo showed lower activity (Figure 6).

It is worth noting that the parent natural zeolite had no antibacterial activity, however some increase in the CFU was observed [43]. An increase in the number of colonies on silver-free clinoptilolite was also found by other authors and it could be explained by better conditions for growth of microorganisms attached on the zeolite surface [52].

According to the MIC results and the activity in the concentration range, the nanomaterials exhibited variable antibacterial properties. The sample Ag–NaZeo was found to be the most effective followed by Ag–Zeo, whereas Ag–HZeo demonstrated a 6-fold lower activity. We assume that the observed difference comes from different Ag content in the nanocomposites. According to the EDX results, the lowest Ag loading was in H-zeolite (Ag 2.2 wt%), while it was higher in Ag–Zeo and Ag–NaZeo, respectively 10.4 and 14.6 wt% (Table 1).

The MIC values with respect to *E. coli* found in this work were in the range of values reported by Hanim et al. for Ag-exchanged zeolite NaY and estimated as 2 mg zeolite/mL in 0.9% saline solution and 0.05 mg zeolite/mL in distilled water [53]. Ferreira et al. determined the MIC for silver-modified faujasite zeolites against *E. coli* at concentrations of 0.2 mg/mL and 0.3 mg/mL [23]. The antibacterial properties of silver-loaded natural zeolites against Gram-positive and Gram-negative bacteria were studied and reported by other authors as well [18,19,23,30,54].

## 4. Conclusions

The comparison of the structural parameters and composition of silver–zeolite materials (Ag–Zeo, Ag–NaZeo and Ag–HZeo) revealed the effect of exchangeable cations in the precursors (natural, Na-modified, H-modified zeolites) on the Ag ion load and on the AgNP fraction formed on the zeolite. The modification of natural zeolite with HCl and NaCl introduced variations in adsorptive and ion-exchange properties of the zeolite framework, that allowed the control of the Ag content and Ag distribution via the change in ion-exchange capability of the parent zeolite. The highest Ag uptake demonstrated Na-modified zeolite, followed by natural zeolite, and the lowest Ag content had H-modified zeolite. As was expected, Na-modified zeolite provided a large number of specific ion-exchange sites for the immobilization of Ag^+^ ions, thus ensuring a higher cation exchange capacity. After heating at 400 °C in air, evenly distributed Ag^+^ ions in zeolite channels underwent nucleation to Ag clusters that aggregated to AgNPs and migrated onto the surface of the zeolite crystals. In both samples, Ag-containing natural zeolite, Ag–Zeo and Ag–loaded Na-modified zeolite, Ag–NaZeo, nanoparticles with a 1–14 nm diameter were observed, while in silver-H-modified zeolite, Ag–HZeo the AgNPs grew up to 10–42 nm. The UV-vis and XPS data revealed that, in zeolite, AgNPs co-existed with significant portion of Ag^+^ ions which remained unreduced at elevated temperature in the presence of air. The Ag–loaded nanocomposites showed strong antibacterial activity against the Gram-negative bacterium *E. coli*. The MIC for Ag–Zeo and Ag–NaZeo was found to be 0.8 mg/mL and for Ag–HZeo 5.0 mg/mL. The overall performance of Ag–NaZeo against *E. coli* was greatest among the nanocomposites. The results of this study clearly showed that natural and Na-modified zeolites can be ion-exchanged with Ag^+^ ions and thermally stabilized to acquire antibacterial properties; thus, such materials can be considered as promising candidates as antibacterial agents.

## Figures and Tables

**Table 1:** Element composition (in wt.%) for all samples based on EDX analysis.

Sample	Si	Al	K	Ca	Mg	Fe	O	Ag
Ag–Zeo	30.6	5.3	2.3	1.4	1.2	1.2	47.6	10.4 ^1^
Ag–NaZeo	29.8	5.2	2.4	1.2	-	0.9	45.9	14.6
Ag–HZeo	36.5	2.4	0.8	1.3	-	1.3	55.5	2.2

^1^ reported data are mean values of EDX scanning at four points.

**Table 2:** Specific surface area and pore size for samples Ag–Zeo, Ag–NaZeo, Ag–HZeo and their corresponding precursors.

Sample	Specific Surface Area (S_BET_*,* m^2^/g)	Micropore Surface Area (S_micro_, m^2^/g)	External Surface Area (S_ext_, m^2^/g)	Total Pore Volume (V_total_, cm^3^/g)	Total Micropore Volume (V_micro_, cm^3^/g)	Average Pore Diameter (D_av_, nm)
Natural Zeolite	19	7	12	0.08	0.003	16
Na-modified Zeolite	18	2	16	0.10	0.001	21
H-modified Zeolite	184	139	45	0.17	0.06	3.7
Ag–Zeo	15	0	15	0.08	0.00	21.0
Ag–NaZeo	15	0	15	0.08	0.00	22.5
Ag–HZeo	90	59	31	0.14	0.03	6.1

**Table 3:** XPS parameters for samples Ag–Zeo, Ag–NaZeo, Ag–HZeo.

Sample	Binding Energy (eV) Photoelectron Line		Binding Energy (eV) Auger Line	Kinetic Energy (eV)	Auger Parameters	Assignment
	Ag3d_5/2_	Ag3d_3/2_	AgM_4_NN		(α’)	
Ag–Zeo	369.4	375.4	1131.0	355.6	725.0	Ag(0)
			1133.0	353.6	723.0	Ag(I)
Ag–NaZeo	369.4	375.4	1131.0	355.6	725.0	Ag(0)
			1133.0	353.6	723.0	Ag(I)
Ag–HZeo	369.0	375.0	1131.0	355.6	725.0	Ag(0)
			1133.0	353.6	723.0	Ag(I)

## Data Availability

The data presented in this study are available upon request by contact with the corresponding author.

## Supplementary Materials

The following are available online at https://www.mdpi.com/article/10.3390/ma14154153/s1, Figure S1: SEM micrographs of silver-zeolite composites: (a) Ag–Zeo, (b) Ag–NaZeo, (c) Ag–HZeo, Figure S2: Adsorption-desorption isotherms for samples: (a) Ag–Zeo, (b) Ag–NaZeo and (c) Ag–HZeo, Figure S3: XRD patterns of silver-zeolite composites Ag–Zeo, Ag–NaZeo, Ag–HZeo, Figure S4: AgMNN Auger spectra of samples Ag–Zeo, Ag–NaZeo, Ag–HZeo, Figure S5: Antibacterial activity of silver-zeolite composites at concentration 5.0 mg/mL: (a) control sample, (b) Ag–NaZeo, (c) Ag–Zeo, (d) Ag–HZeo, Figure S6: Antibacterial activity of silver-zeolite composites at concentration 3.0 mg/mL: (a) Ag–Zeo, (b) Ag–NaZeo, (c) Ag–HZeo, Figure S7: Antibacterial activity of Ag–Zeo (upper row) and Ag–NaZeo (lower row) at concentrations of 1.0, 0.8 and 0.6 mg/mL (from left to right).

## References

[1] Baerlocher C., McCusker L.B., Olsen D.H. (2007). Atlas of Zeolite Framework Types.

[2] https://europe.iza-structure.org/IZA-SC/framework_3d.php?STC=HEU.

[3] Koyoma K., Takeuchi Y. (1977). Clinoptilolite: The distribution of potassium atoms and its role in thermal stability. Z. Krist..

[4] Ünaldi T., Kadir S., Kilislioglu A. (2012). Unheated and Heated Batch Methods in Ion Exchange of Clinoptilolite. Ion Exchange Technologies.

[5] Pansini M. (1996). Natural zeolites as cation exchangers for environmental protection. Mineral. Depos..

[6] Margeta K., Logar N.Z., Šiljeg M., Farkaš A., Elshorbagy W., Chowdhury R. (2013). Natural Zeolites in Water Treatment—How Effective is Their Use. Water Treatment.

[7] Nakhli S.A.A., Delkash M., Bakhshayesh B.E., Kazemian H. (2017). Application of Zeolites for Sustainable Agriculture: A Review on Water and Nutrient Retention. Water Air Soil Pollut..

[8] Pavelić S.K., Medica J.S., Gumbarević D., Filošević A., Pržulj N., Pavelić K. (2018). Critical Review on Zeolite Clinoptilolite Safety and Medical Applications in vivo. Front. Pharmacol..

[9] Servatan M., Zarrintaj P., Mahmodi G., Kim S.-J., Ganjali M.R., Saeb M.R., Masoud Mozafari M. (2020). Zeolites in Drug Delivery: Progress, Challenges and Opportunities. Drug Discov. Today.

[10] Sprynskyy M., Buszewski B., Terzyk A.P., Namieśnik J. (2006). Study of the selection mechanism of heavy metal (Pb^2+^, Cu^2+^, Ni^2+^, and Cd^2+^) adsorption on clinoptilolite. J. Colloid. Interface Sci..

[11] Fu H., Li Y., Yu Z., Shen J., Li J., Zhang M., Ding T., Xu L., Lee S.S. (2020). Ammonium removal using a calcined natural zeolite modified with sodium nitrate. J. Hazard. Mater..

[12] Inglezakis V.J., Satayeva A., Yagofarova A., Tauanov Z., Meiramkulova K., Farrando-Pérez J., Bear J.C. (2020). Surface Interactions and Mechanisms Study on the Removal of Iodide from Water by Use of Natural Zeolite-Based Silver Nanocomposites. Nanomaterials.

[13] Huang Y., Su W., Wang R., Zhao T. (2019). Removal of Typical Industrial Gaseous Pollutants: From Carbon, Zeolite, and Metal-organic Frameworks to Molecularly Imprinted Adsorbents. Aerosol Air Qual. Res..

[14] Bacariza M.C., Graça I., Lopes J.M., Henriques C. (2019). Tuning zeolite properties towards CO_2_ methanation: An overview. ChemCatChem.

[15] Panayotova M.I. (2001). Kinetics and thermodynamics of copper ions removal from wastewater by use of zeolite. Waste Manage..

[16] Znak Z.O., Kornii S.A., Mashtaler A.S., Zin O.I. (2021). Production of Nanoporous Zeolites Modified by Silver Ions with Antibacterial Properties. Mater. Sci..

[17] Hao J., Lang S., Mante F., Pavelić K., Ozer F. (2021). Antimicrobial and Mechanical Effects of Zeolite Use in Dental Materials: A Systematic Review. Acta Stomatol. Croat..

[18] Dutta P., Wang B. (2019). Zeolite-supported silver as antimicrobial agents. Coord. Chem. Rev..

[19] Otávio de Araújo L., Anaya K., Pergher S.B.C. (2019). Synthesis of Antimicrobial Films Based on Low-Density Polyethylene (LDPE) and Zeolite A Containing Silver. Coatings.

[20] Nagy A., Harrison A., Sabbani S., Munson R.S., Dutta P., Waldman W.J. (2011). Silver nanoparticles embedded in zeolite membranes: Release of silver ions and mechanism of antibacterial action. Int. J. Nanomed..

[21] Jiraroj D., Tungasmita S., Tungasmita D.N. (2014). Silver Ions and Silver Nanoparticles in Zeolite A Composites for Antibacterial Activity. Powder Technol..

[22] Rivera-Garza M., Olguín M.T., García-Sosa I., Alcántara D., Rodríguez-Fuentes G. (2000). Silver supported on natural Mexican zeolite as an antibacterial material. Microporous Mesoporous Mater..

[23] Ferreira L., Fonseca A.M., Botelho G., Almeida-Aguiar C., Neves I.C. (2012). Antimicrobial activity of faujasite zeolites doped with silver. Microporous Mesoporous Mater..

[24] Demirci S., Ustaoğlu Z., Yılmazer G.A., Sahin F., Baç N. (2014). Antimicrobial Properties of Zeolite-X and Zeolite-A Ion-Exchanged with Silver, Copper, and Zinc Against a Broad Range of Microorganisms. Appl. Biochem. Biotechnol..

[25] Yamanaka M., Hara K., Kudo J. (2005). Bactericidal Actions of a Silver Ion Solution on *Escherichia coli*, Studied by Energy-Filtering Transmission Electron Microscopy and Proteomic Analysis. Appl. Environ. Microbiol..

[26] Shameli K., Ahmad M.B., Zargar M., Yunus W.M., Ibrahim N.A. (2011). Fabrication of silver nanoparticles doped in the zeolite framework and antibacterial activity. Int. J. Nanomed..

[27] Concepción-Rosabal B., Rodríguez-Fuentes G., Bogdanchikova N., Bosch P., Avalos M., Lara V.H. (2005). Comparative study of natural and synthetic clinoptilolites containing silver in different states. Microporous Mesoporous Mater..

[28] Flores-López N.S., Castro-Rosas J., Ramírez-Bon R., Mendoza-Córdova A., Larios-Rodríguez E., Flores-Acosta M. (2012). Synthesis and properties of crystalline silver nanoparticles supported in natural zeolite chabazite. J. Mol. Str..

[29] Yee M.S.-L., Khiew P.S., Tan Y.F., Chiu W.S., Kok Y.-Y., Leong C.-O. (2015). Low temperature, rapid solution growth of antifouling silver-zeolite nanocomposite clusters. Microporous Mesoporous Mater..

[30] Azambre B., Chebbi M., Hijazi A. (2020). Effects of the cation and Si/Al ratio on CH_3_I adsorption by faujasite zeolites. Chem. Eng. J..

[31] Panayotova M., Mintcheva N., Gicheva G., Djerahov L., Mirdzveli N. Modified Clinoptilolite as Precursor for Formation of Silver Nanoparticles-Zeolite Nanocomposites. In Proceedings of 20th International Multidisciplinary Scientific GeoConference SGEM 2020.

[32] Inglezakis V.J., Loizidou M.M., Grigoropoulou H.P. (2004). Ion Exchange Studies on Natural and Modified Zeolites and the Concept of Exchange Site Accessibility. J. Colloid Interface Sci..

[33] Top A., Ülkü S. (2004). Silver, Zinc, and Copper Exchange in a Na-Clinoptilolite and Resulting Effect on Antibacterial Activity. Appl. Clay Sci..

[34] Lihareva N., Dimova L., Petrov O., Tzvetanova Y. (2010). Ag+ sorption on natural and Na-exchanged clinoptilolite from Eastern Rhodopes, Bulgaria. Microporous Mesoporous Mater..

[35] Sing K.S. (1985). Reporting physisorption data for gas/solid systems with special reference to the determination of surface area and porosity (recommendations 1984). Pure Appl. Chem..

[36] Sing K.S.W., Williams R.T. (2004). Physisorption Hysteresis Loops and the Characterization of Nanoporous Materials. Adsorpt. Sci. Technol..

[37] Atalay B., Gündüz G. (2011). Isomerizaton of α-Pinene over H3PW12O40 Catalysts Supported on Natural Zeolite. Chem. Eng. J..

[38] Sprynskyy M., Golembiewski R., Trykowski G., Buszewski B. (2010). Heterogeneity and Hierarchy of Clinoptilolite Porosity. J. Phys. Chem. Solids.

[39] Dziedzicka A., Sulikowski B., Ruggiero-Mikołajczyk M. (2015). Catalytic and Physicochemical Properties of Modified Natural Clinoptilolite. Catal. Today.

[40] Wang C., Leng S., Guo H., Cao L., Huang J. (2019). Acid and Alkali Treatments for Regulation of Hydrophilicity/Hydrophobicity of Natural Zeolite. Appl. Surf. Sci..

[41] Mondloch J.E., Bayram E., Finke R.G. (2012). A review of the kinetics and mechanisms of formation of supported-nanoparticle heterogeneous catalysts. J. Mol. Catal. A Chem..

[42] Bartolomeu R., Bértolo R., Casale S., Fernandes A., Henriques C., Da Costa P., Ribeiro F. (2013). Particular characteristics of silver species on Ag-exchanged LTL zeolite in K and H form. Microporous Mesoporous Mater..

[43] Panayotova M.I., Mintcheva N.N., Gemishev O.T., Tyuliev G.T., Gicheva G.D., Djerahov L.P. (2018). Preparation and Antimicrobial Properties of Silver Nanoparticles Supported by Natural Zeolite Clinoptilolite. Bulg. Chem. Comm..

[44] Rodríguez Iznaga I., Petranovskii V., Rodríguez Fuentes G., Mendoza C., Benítez Aguilar A. (2007). Exchange and reduction of Cu^2+^ ions in clinoptilolite. J. Colloid Interf. Sci..

[45] Sydorchuk V., Vasylechko V., Khyzhun O., Gryshchouk G., Khalameida S., Vasylechko L. (2021). Effect of high-energy milling on the structure, some physicochemical and photocatalytic properties of clinoptilolite. Appl. Catal. A Gen..

[46] Rodríguez-Iznaga I., Petranovskii V., Castillón-Barraza F., Concepción-Rosabal B. (2011). Copper-silver bimetallic system on natural clinoptilolite: Thermal reduction of Cu^2+^ and Ag^+^ exchanged. J. Nanosci. Nanotechnol..

[47] Bartolomeu R., Azambre B., Westermann A., Fernandes A., Bertolo R., Issa Hamoud H., Henriques C., Da Costa P., Ribeiro F. (2014). Investigation of the nature of silver species on different Ag-containing NOx reduction catalysts: On the effect of the support. Appl. Catal. B Environ..

[48] Greczynski G., Hultman L. (2020). Compromising Science by Ignorant Instrument Calibration-Need to Revisit Half a Century of Published XPS Data. Angew. Chem. Int. Ed. Engl..

[49] Moulder J.F., Stickle W.F., Sobol P.E., Bomben K.D., Chastain J. (1992). Handbook of X-ray Photoelectron Spectroscopy.

[50] Fonseca M.A.M., Neves I.C. (2013). Study of silver species stabilized in different microporous zeolites. Microporous Mesoporous Mater..

[51] Childs K.D., Carlson B.A., Vanier L.A., Moulder J.F., Paul D.F., Stickle W.F., Watson D.G., Hedberg C.L. (1995). Handbook of Auger Electron Spectroscop.

[52] Guerra R., Lima E., Viniegra M., Guzmán A., Lara V. (2012). Growth of Escherichia coli and Salmonella typhi inhibited by fractal silver nanoparticles supported on zeolites. Microporous Mesoporous Mater..

[53] Hanim S.A.M., Malek N.A.N.N., Ibrahim Z. (2016). Amine-functionalized, silver-exchanged zeolite NaY: Preparation, characterization and antibacterial activity. Appl. Surf. Sci..

[54] Kędziora A., Speruda M., Krzyżewska E., Rybka J., Łukowiak A., Bugla-Płoskońska G. (2018). Similarities and Differences between Silver Ions and Silver in Nanoforms as Antibacterial Agents. Int. J. Mol. Sci..

