# Supplementary material for: Effect of Exchangeable Ions in Natural and Modified Zeolites on Ag Content, Ag Nanoparticle Formation and Their Antibacterial Activity
Source: Materials (Basel). 2021 Jul 26;14(15):4153. doi: 10.3390/ma14154153 (PMC8347238; doi:10.3390/ma14154153)
Supplement: Supplementary file 1 [file materials-14-04153-s001.zip › materials-1277769-supplementary.pdf]

materials

Supplementary Materials

Effect of Exchangeable Ions in Natural and Modified Zeolites on Ag Content, Ag Nanoparticle Formation and Their Antibacterial Activity

Neli Mintcheva [1,*], Marinela Panayotova [1], Gospodinka Gicheva [1], Orlin Gemishev [2] and Georgy Tyuliev [3]

1 Department of Chemistry, University of Mining and Geology, 1700 Sofia, Bulgaria; marichim@mgu.bg (M.P.); g.gicheva@mgu.bg (G.G.)
2 Faculty of Biology, Sofia University "St. Kliment Ohridski", 1000 Sofia, Bulgaria; o.gemishev@uni-sofia.bg
3 Institute of Catalysis, Bulgarian Academy of Sciences, 1113 Sofia, Bulgaria; tyuliev@ic.bas.bg
* Correspondence: nnmintcheva@mgu.bg

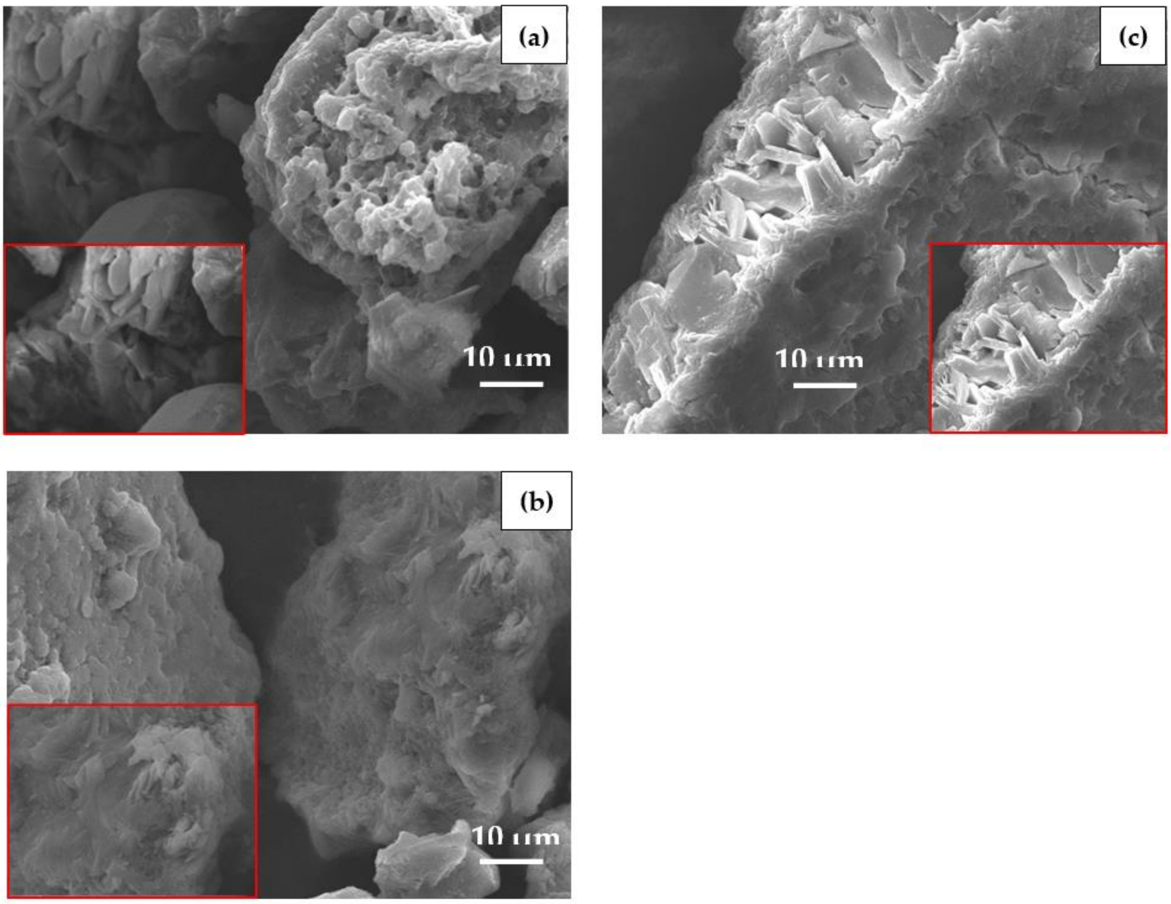

Figure S1. SEM micrographs of silver-zeolite composites: (**a**) Ag–Zeo, (**b**) Ag–NaZeo, (**c**) Ag–HZeo.

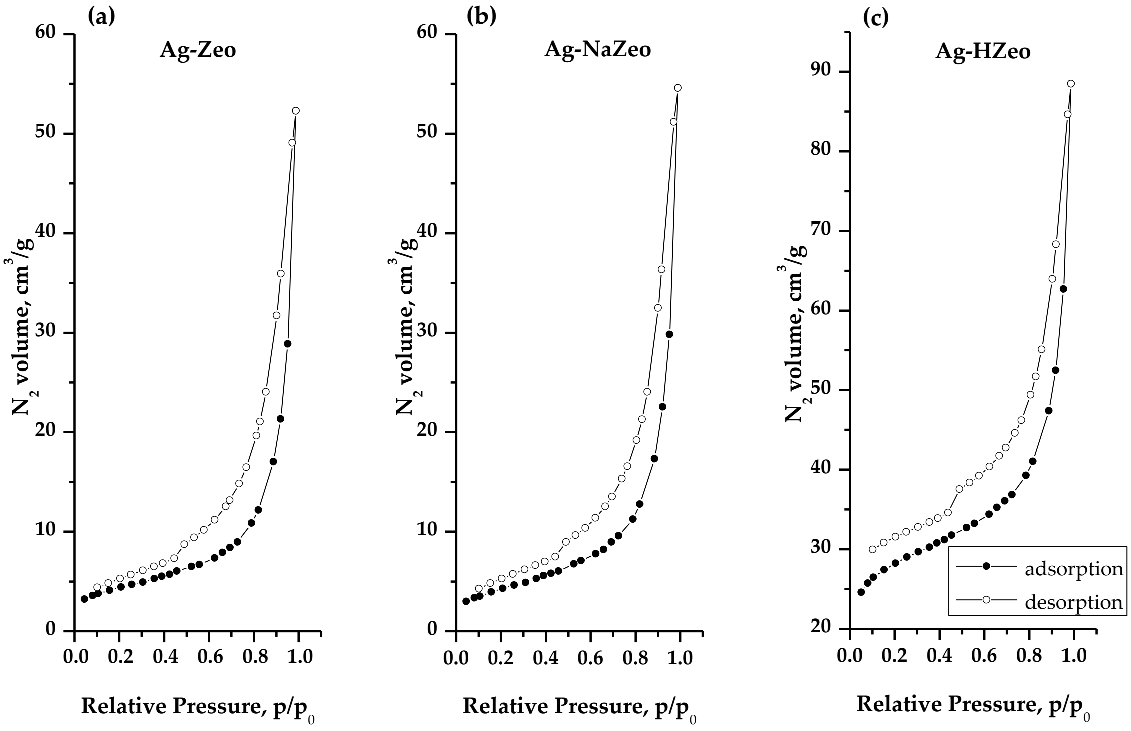

Figure S2. Adsorption-desorption isotherms for samples: (**a**) Ag–Zeo, (**b**) Ag–NaZeo and (**c**) Ag–HZeo.

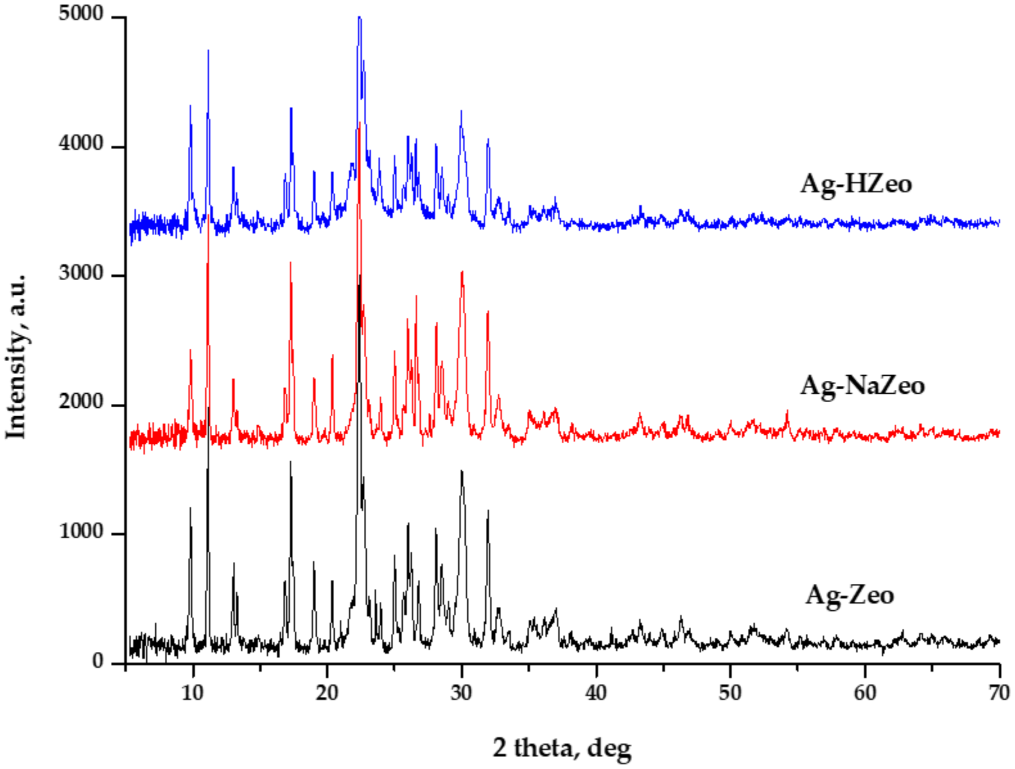

Figure S3. XRD patterns of silver-zeolite composites Ag–Zeo, Ag–NaZeo, Ag–HZeo.

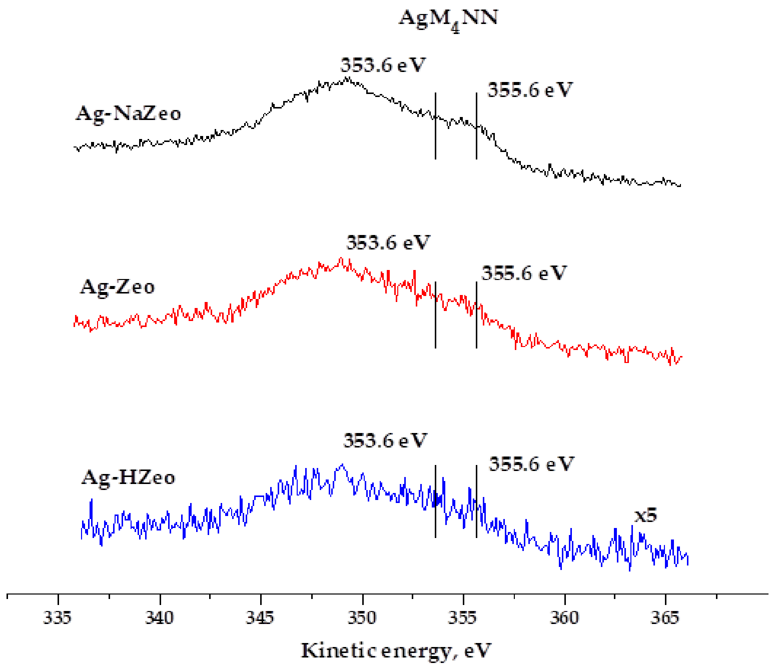

Figure S4. AgMNN Auger spectra of samples Ag–Zeo, Ag–NaZeo, Ag–HZeo.

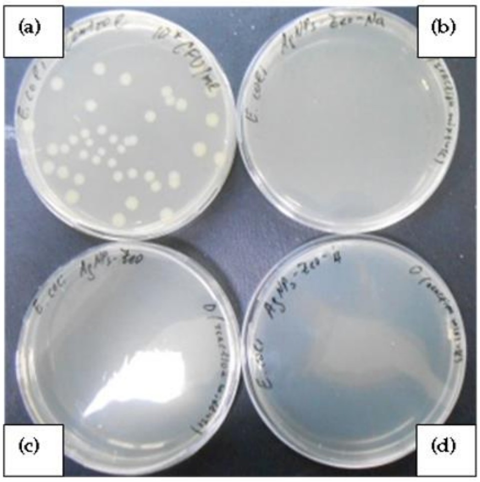

Figure S5. Antibacterial activity of silver-zeolite composites at concentration 5.0 mg/mL: (**a**) control sample, (**b**) Ag–NaZeo, (**c**) Ag–Zeo, (**d**) Ag–HZeo.

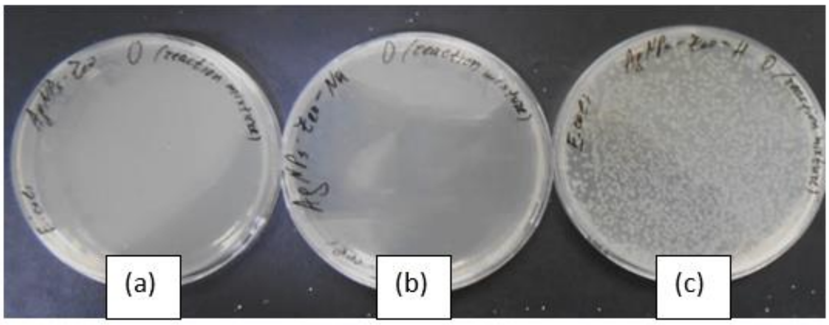

Figure S6. Antibacterial activity of silver-zeolite composites at concentration 3.0 mg/mL: (**a**) Ag–Zeo, (**b**) Ag–NaZeo, (**c**) Ag–HZeo.

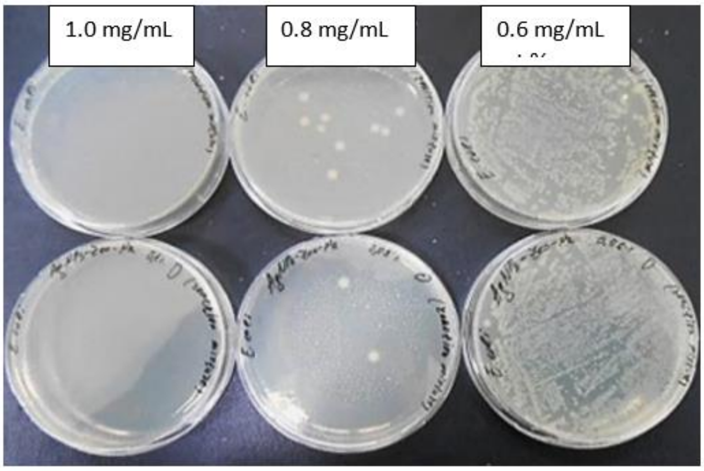

Figure S7. Antibacterial activity of Ag–Zeo (upper row) and Ag–NaZeo (lower row) at concentrations of 1.0, 0.8 and 0.6 mg/mL (from left to right).
